# Metabolic signatures of germination triggered by kinetin in *Medicago truncatula*

**DOI:** 10.1038/s41598-019-46866-6

**Published:** 2019-07-18

**Authors:** Susana Araújo, Andrea Pagano, Daniele Dondi, Simone Lazzaroni, Eduardo Pinela, Anca Macovei, Alma Balestrazzi

**Affiliations:** 1 Instituto de Tecnologia Química e Biológica António Xavier - Universidade Nova de Lisboa (ITQB-NOVA), Av. da República, 2780-157 Oeiras, Portugal; 2 Department of Biology and Biotechnology ‘L. Spallanzani’, via Ferrata 9, 27100 Pavia, Italy; 3 Department of Chemistry, Viale Taramelli 12, 27100 Pavia, Italy

**Keywords:** Systems biology, Plant sciences

## Abstract

In the present work, non-targeted metabolomics was used to investigate the seed response to kinetin, a phytohormone with potential roles in seed germination, still poorly explored. The aim of this study was to elucidate the metabolic signatures of germination triggered by kinetin and explore changes in metabolome to identify novel vigor/stress hallmarks in *Medicago truncatula*. Exposure to 0.5 mM kinetin accelerated seed germination but impaired seedling growth. Metabolite composition was investigated in seeds imbibed with water or with 0.5 mM kinetin collected at 2 h and 8 h of imbibition, and at the radicle protrusion stage. According to Principal Component Analysis, inositol pentakisphosphate, agmatine, digalactosylglycerol, inositol hexakisphosphate, and oleoylcholine were the metabolites that mostly contributed to the separation between 2 h, 8 h and radicle protrusion stage, irrespective of the treatment applied. Overall, only 27 metabolites showed significant changes in mean relative contents triggered by kinetin, exclusively at the radicle protrusion stage. The observed metabolite depletion might associate with faster germination or regarded as a stress signature. Results from alkaline comet assay, highlighting the occurrence of DNA damage at this stage of germination, are consistent with the hypothesis that prolonged exposure to kinetin induces stress conditions leading to genotoxic injury.

## Introduction

At the onset of germination, the transition from a dry quiescent state to a fully hydrated state is associated with extensive metabolic changes^1,2^. This is a crucial phase for seed viability and vigor since water up-take causes increased ROS (reactive oxygen species)-mediated oxidative injury^3,4^, and exacerbates genotoxic damage^5,6^. These deleterious effects are counterbalanced by effective ROS scavenging mechanisms that limit the damage within the cellular environment^7^ whereas the removal of DNA lesions is carried by dedicated repair pathways that contribute to safeguard genome integrity^8–11^. The successful mitigation of oxidative damage towards macromolecules, including genotoxic insults correlates with enhanced seed vigor^11,12^. DNA repair represents a distinctive feature of the seed pre-germinative metabolism and the work by Waterworth *et al*.^6^ has clearly highlighted the crucial role of the DNA checkpoint kinase ATM (ATAXIA TELANGIECTASIA MUTATED) and ATR (ATM AND RAD3-RELATED) as major determinants of seed viability and vigor in the model plant *Arabidopsis thaliana*. Studies carried in the model legume *Medicago truncatula* have demonstrated that the DNA damage response triggered during early seed imbibition features the up-regulation of key BER (base excision repair) genes, as *OGG1* (8-oxoguanine glycosylase/lyase) and *FPG* (formamidopyrimidine DNA glycosylase) required to remove the DNA oxidative lesions under physiological and stress conditions^9,10,13^.

The positive correlation between an effective antioxidant and DNA repair response within the pre-germinative metabolism and the degree of seed vigor is the driving force of priming, a pre-sown technique that allows to carry seed imbibition under controlled conditions, in order to boost the seed repair and protective activities^14–16^. Additional benefits can rise by adding bioactive molecules, e.g. phytohormones, however the treatment must be stopped before radicle protrusion occurs otherwise desiccation tolerance will be lost^17,18^. In the case of ‘hormopriming’, phytohormones are added to boost germination and seedling robustness, as in the case of gibberellic acid that promotes antioxidant response and abiotic stress tolerance in white clover and wheat^19^.

Exogenous applications of phytohormones generally ameliorate the stress response *in planta*, allowing to better regulate and integrate the growth process in changing environments. Cytokinins have been implicated in fruit and seed development^20^ and transgenic plants overexpressing the *ipt* (isopentenyl transferase) gene, essential for cytokinin biosynthesis, were characterized by increased seed yield and quality^20^. Initial studies revealed the ‘permissive’ role of cytokinins in seed germination^21^ while more recently it has been reported that, at the onset of germination, cytokinin counteracts the inhibitory role of ABA by downregulating the expression of the *ABI5* (*ABA INSENSITIVE*) gene^22^. The effects of cytokinins, including kinetin (6-furfurylaminopurine), as priming agents to improve seed germination and seedling robustness have been described and possibly related to enhanced cell division rates^23,24^. As for legumes, cytokinins are engaged in the intricated networking underlying symbiotic nodulation, as shown in the model plant *M*. *truncatula*^25^ and have been included, among other phytohormones, in culture media formulations tailored for *in vitro* regeneration via somatic embryogenesis. However, information about the effects of kinetin in the context of seed germination, namely about the potential use of this growth regulator to stimulate germination, is still scanty. In this context, legumes represent an attractive system to explore and assess the possible benefits of kinetin-mediated priming. A major issue of grain legumes is the need to stabilize yields in water-limited environments since, compared to other crops, they have higher water requirements. Legumes are used in rotational and intercropping systems during the post-rainy season or in lands unsuitable for the main crop and the availability of seeds primed by controlled hydration before sown improves seedling emergence and establishment^26^. Indeed, reduced field performance generally results from the variability in number and speed of seedling emergence, thus reflecting poor seed vigor^27^. These unfavorable features can be mitigated by means of tailored priming protocols that require, however, a deeper technical knowedge tightly linked to a better comprehension of the molecular aspects of the pre-germinative metabolism^28^.

Metabolomics studies are very useful approaches to retrieve overall metabolites differentially accumulated during the different germination phases and possibly linked to seed vigor^13,29–31^. The aim of the present study was to elucidate the physiological and metabolic signatures of *M*. *truncatula* seed germination in response to exogenously applied kinetin. Besides being a reference legume for functional biology, and for the study of the rhizobia-legume symbiosis as well as the integrated stress and growth responses, *M*. *truncatula* is also a pasture species used first in Australia in the wheat/sheep rotation^32^ and more recently in South Africa in rotation with wheat, barley and canola^33^. As for other pulses, *M*. *truncatula* seed and seedling vigor is among the selection criteria used in breeding programmes finalized to the development of novel annual medics^34^. In the attempt to improve germination performance and stand establishment, a further step is required, namely a deeper dissection of the molecular determinants of seed vigor that might possibly include metabolites able to alleviate genotoxic stress. In this work, a multidisciplinary approach, combining non-targeted metabolite profiling, gene expression analysis, electron paramagnetic resonance, and comet assay has been used to gain an in-depth picture of the response to kinetin in legume seeds. Attention was focused on the impact of exogenously applied kinetin on seed germination as well as on the stress conditions resulting from prolonged exposure to the phytohormone. This work contributes to the current state of the art regarding the issues of seed vigor in legumes, and adds another piece of information useful to decipher the complex molecular network and specific hallmarks underlying the seed response to stress. Due to the high synteny of this model species with other legumes, the results of this investigation can impact research on seed biology in other economically relevant legume crops, such as pulses.

## Results

### Kinetin promotes seed germination while impairing root growth

To select the most suitable kinetin dose, preliminary work was carried out using 0.25, 0.5, and 0.75 mM kinetin. As shown in Supplementary Fig. 1, a significant increase (*P* < 0.01) in germination percentage was observed in response to 0.5 mM and 0.75 mM kinetin, with estimated values of 72.00 ± 15.24% (0.5 mM kinetin) and 76.00 ± 15.57% (0.75 mM kinetin), respectively. Additionally, the T_50_ parameter (number of days required to reach half maximal germination, see Materials and Methods) was significantly reduced when comparing the 0.5 mM and 0.75 mM kinetin doses with control samples imbibed with water (W) (Supplementary Table 1). On the other hand, inhibition of root growth was observed in four-day-old *M*. *truncatula* seedlings developed in presence of kinetin (Supplementary Table 1 and Supplementary Fig. 2). The lowest dose able to anticipate germination, 0.5 mM kinetin (hereby named Kin), was selected for further investigation.

**Figure 1:**
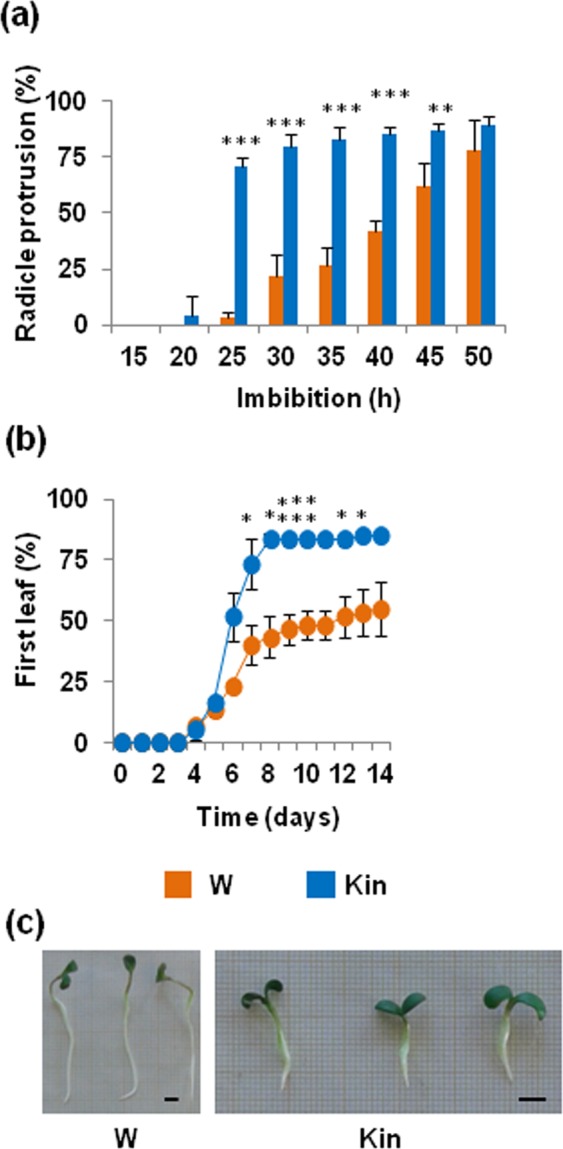
Impact of kinetin on seed germination in *M*. *truncatula*. Imbibition with 0.5 mM kinetin (Kin) accelerates radicle protrusion (**a**) and anticipates the occurrence of the first true leaf (**b**), compared to control seeds imbibed with water (W). (**c**) Phenotype of four-day-old *M*. *truncatula* seedlings developed from seeds imbibed with water (W) and 0.5 mM kinetin (Kin), respectively. Values are expressed as mean ± SD of three independent replications with 20 seeds for each replication. Asterisks indicate statistically significant differences determined using Student’s *t*-test (*P < *0.05). Bar = 0.5 cm.

The Kin treatment resulted in accelerated radicle emergence (Fig. 1a) and anticipation of the first true leaf appearance was also observed (Fig. 1b). The phenotype of four-day-old *M*. *truncatula* seedlings developed from seeds imbibed with 0.5 mM kinetin is shown in Fig. 1c. The growth impairment caused by prolonged exposure to kinetin is particularly evident at the root level. The 0.5 mM concentration was chosen among the tested ones because this was the lowest dose that could anticipate germination. In view of further applications of these results to design new priming protocols, this also represents the most cost-effective concentration.

**Figure 2:**
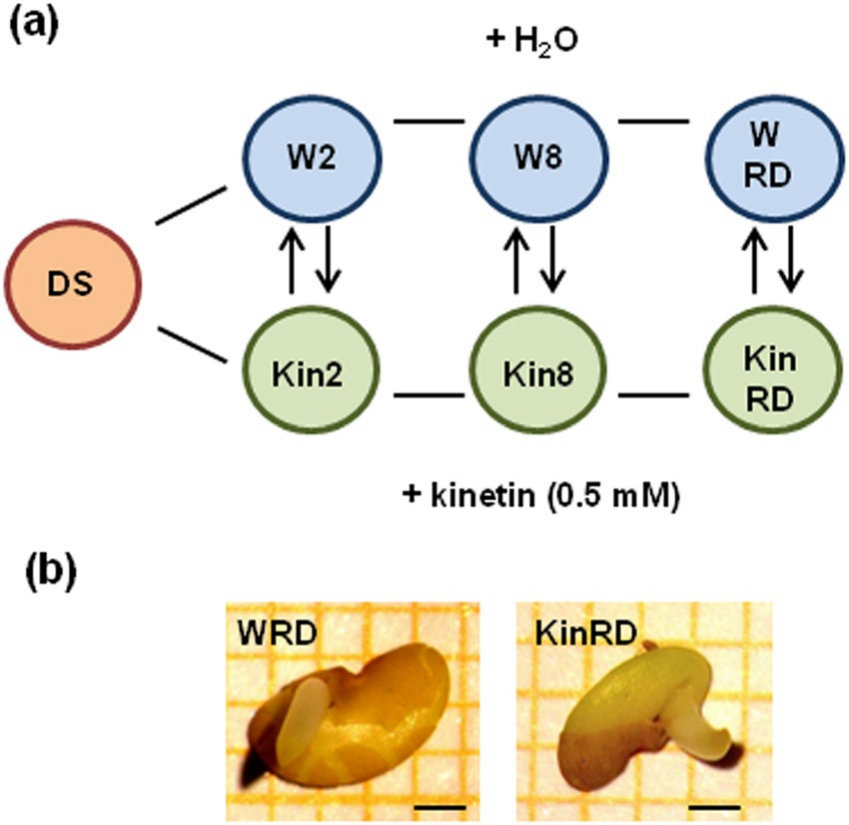
(**a**) Experimental design. Metabolite composition was investigated in *M*. *truncatula* seeds imbibed with water (W) and 0.5 mM kinetin (Kin), respectively, collected at 2 h and 8 h of imbibition, and at the radicle protrusion stage (RD). (**b**) The phenological stage of radicle protrusion occurred faster in kinetin-treated seeds (KinRD) compared to control seeds imbibed with water (WRD). Kinetin-treated seeds were collected starting from the 25^th^ hour from the beginning of imbibition whereas water-treated seeds were collected subsequently, starting from the 40^th^ hours. Bar = 1 mm.

To better understand the molecular mechanisms behind the observed phenotype induced by this treatment, the kinetin-triggered changes in metabolite profiles were investigated. Thereby, the metabolite composition was analyzed in *M*. *truncatula* seeds imbibed with water (W) or with 0.5 mM kinetin (Kin) collected at: (*i*) 2 h (hereby named W2 and Kin2) and (*ii*) 8 h (hereby named W8 and Kin8) of imbibition, as well as at the (*iii*) radicle protrusion stage (radicle length ≤ 3 mm; hereby named WRD and KinRD). With this experimental design (Fig. 2a), attention was focused on two pre-germination timepoints (2 h and 8 h of imbibition), as well as on the developmental stage of radicle protrusion, previously reported to undergo extensive metabolic changes^1,2,13^. Due to the reduced T_50_ value observed in the phytohormone-treated seeds, the sampling procedure for the radicle protrusion stage was conducted earlier than for water-imbibed seeds. Thus, the majority of kinetin-treated seeds were collected starting from the 25^th^ hour from the beginning of imbibition whereas the water-treated seeds were collected subsequently, starting from the 40^th^ hour (Fig. 1a). The phenotype of W and Kin seeds at the radicle protrusion stage is shown in Fig. 2b.

### Kinetin affects nucleotide, amino acid, lipids, and carbohydrate metabolism during germination

**Figure 3:**
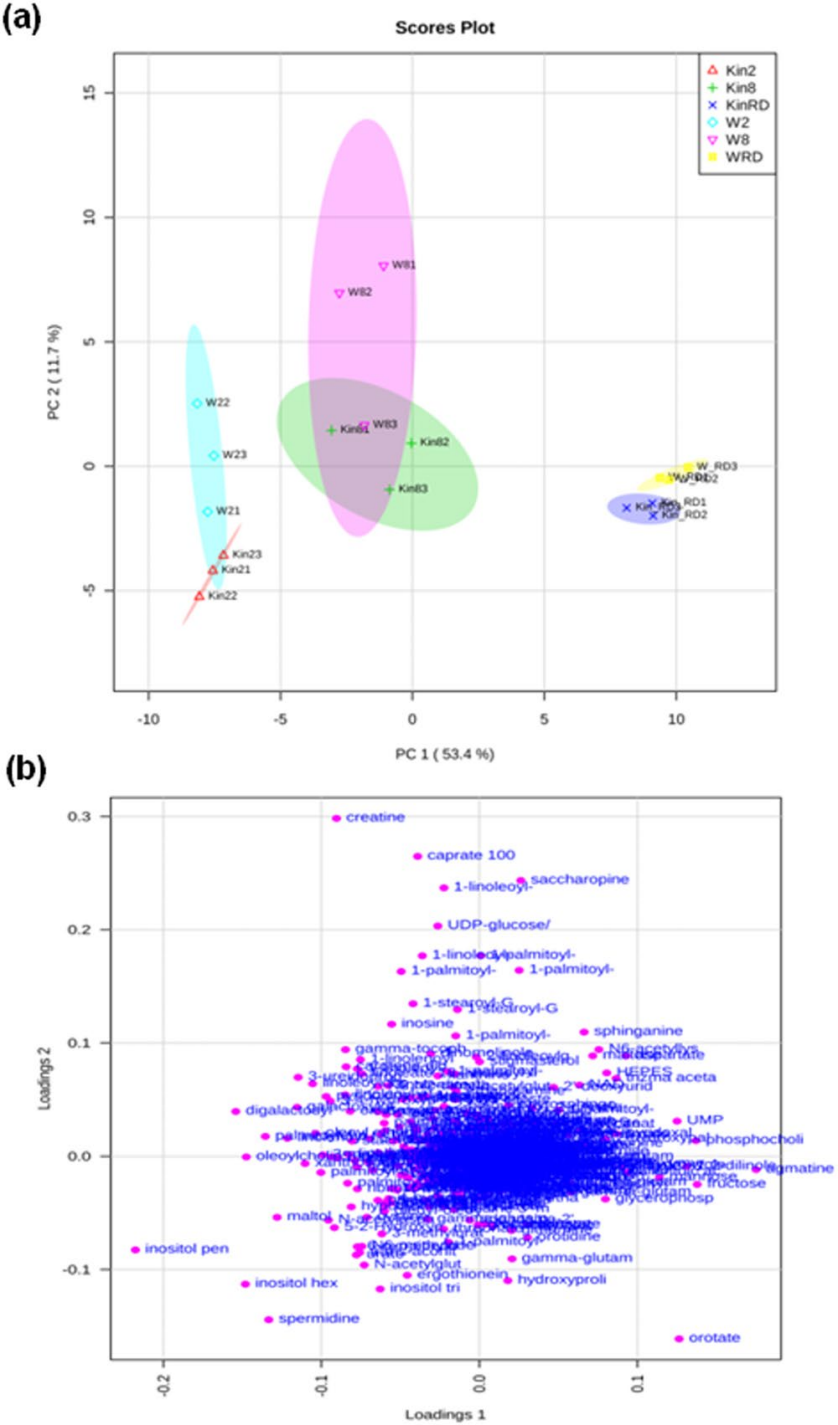
Principal Component Analysis (PCA) of the 411 metabolites detected in response to Kin and W treatments, sampled at 2 h and 8 h of imbibition, and at radicle protrusion stage (RD). The log-transformed raw scaled imputed value for each metabolite in response to experimental conditions was used for computation. (**a**) PCA score plot explaining 65.1% of the variance of metabolite profiles, in which PC1 accounts for 53.4%, while PC2 accounts for 11.7% of the variance. (**b**) Loading plots highlighting the metabolites contributed to the differences observed among samples. W2, seeds imbibed with water for 2 h. W8, seeds imbibed with water for 8 h. WRD, seeds imbibed with water collected at the radicle protrusion stage. Kin2, seeds imbibed with 0.5 mM kinetin for 2 h. Kin8, seeds imbibed with 0.5 mM kinetin for 8 h. KinRD, seeds imbibed with 0.5 mM kinetin collected at the radicle protrusion stage.

A total of 412 metabolites with known structures were identified (Supplementary Dataset S1). Among these, kinetin was only detected in kinetin-treated samples and thus removed from the pool of metabolites to be further studied. A Principal component analysis (PCA) was applied to the 411 metabolites identified in the metabolomic study (Fig. 3) in order to investigate the differences between samples, the similarity between replicates, and which metabolites (variables) contributed mostly to this difference. The two main factors/components extracted accounted for 65.1% of the variance. Principal component one (PC1) accounted for 53.4% of the total variance and, according to the respective loadings plot (Fig. 3a), inositol pentakisphosphate (0.21), agmatine (0.17), digalactosylglycerol (0.15), inositol hexakisphosphate (0.14), and oleoylcholine (−0.14) were the metabolites that mostly contributed to the separation between 2 h, 8 h and radicle protrusion stage, irrespective to the treatment applied. PC2 accounts for 11.7% of the variation and, according to the respective loadings plot (Fig. 3b), creatine (0.29), caprate (0.26), saccharopine (0.24), 1-linoleoyl-GPI 182 (0.23), UDP-glucose/UDP-galactose (0.20), and orotate (−0.13) were the metabolites that mostly contributed to the separation in this axis. No major differences in metabolomic profiles were detected between untreated and kinetin-treated samples at 2 h and 8 h of imbibition since these samples clustered together. Nevertheless, PCA indicated that the effects of kinetin on metabolomic profiles were relevant at the radicle protrusion stage, where the separation of kinetin-treated and untreated samples was unveiled. The univariate analysis allowed to identify 27 metabolites showing significant changes in the mean relative contents triggered by kinetin treatment (herein defined as *P* ≤ 0.05 and *q* ≤ 0.1), exclusively at the radicle protrusion stage (RD) (Supplementary Dataset S1). Changes in the mean metabolite contents in seeds imbibed with kinetin for 2 h and untreated seeds were noticed (7 up- and 26 down-accumulated metabolites) based on *P* ≤ 0.05 but they did not meet the *q* significance threshold imposed (*q* ≥ 0.1). Nevertheless, it is worth to mention the accumulation of deoxycarnitine (FC = 1.82, *P* = 0.001, *q* = 0.115) and 1-palmitoyl-2-linoleoyl-GP (FC = 3.34, *P* = 0.0033, *q* = 0.1531) as well as the depletion of 2-hydroxymyristate (FC = 0.6, *P* = 0.0043, *q* = 0.1651), 5-methylcytidine (FC = 0.8, *P* = 0.003, *q* = 0.1531), and salicylate (FC = 0.8, *P* = 0.0022, *q* = 1531) observed in seeds imbibed for 2 h with kinetin over untreated seeds, which are borderline with the significance threshold imposed. A reduced number of metabolites showing changes in content was noticed (1 up- and 8 down-accumulated metabolites, based on *P* ≤ 0.05) in seeds imbibed with kinetin for 8 h and untreated seeds, although these changes also did not meet the threshold imposed for *q* significance.

**Figure 4:**
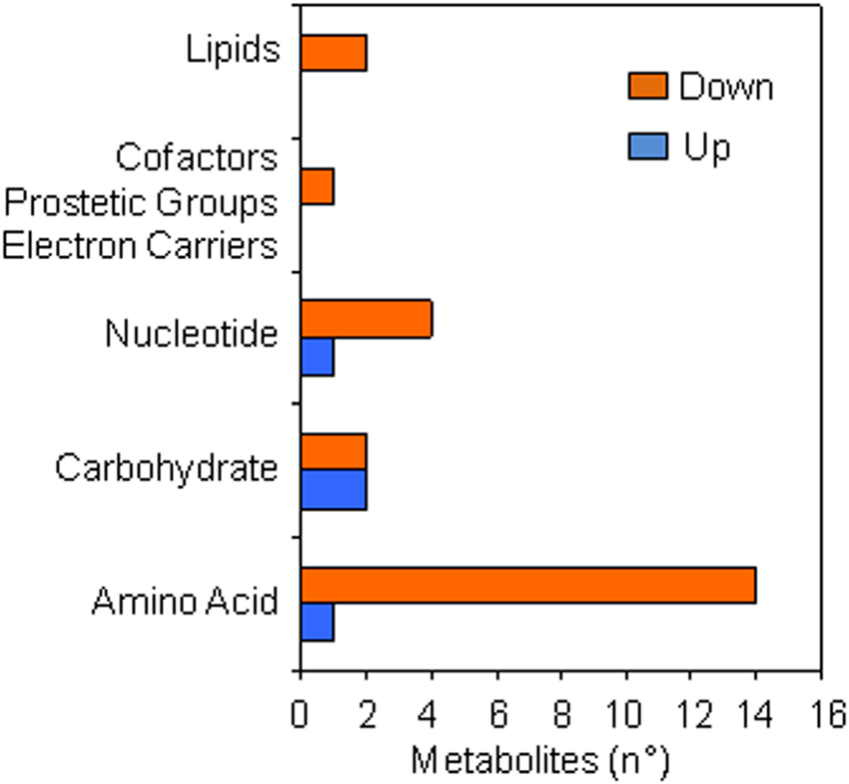
Metabolic super-pathways significantly (*P* ≤ 0.05 and *q* ≤ 0.1) changed in response to kinetin treatment at the radicle protrusion stage compared. The number of metabolites significantly up- and down- accumulated is depicted.

**Figure 5:**
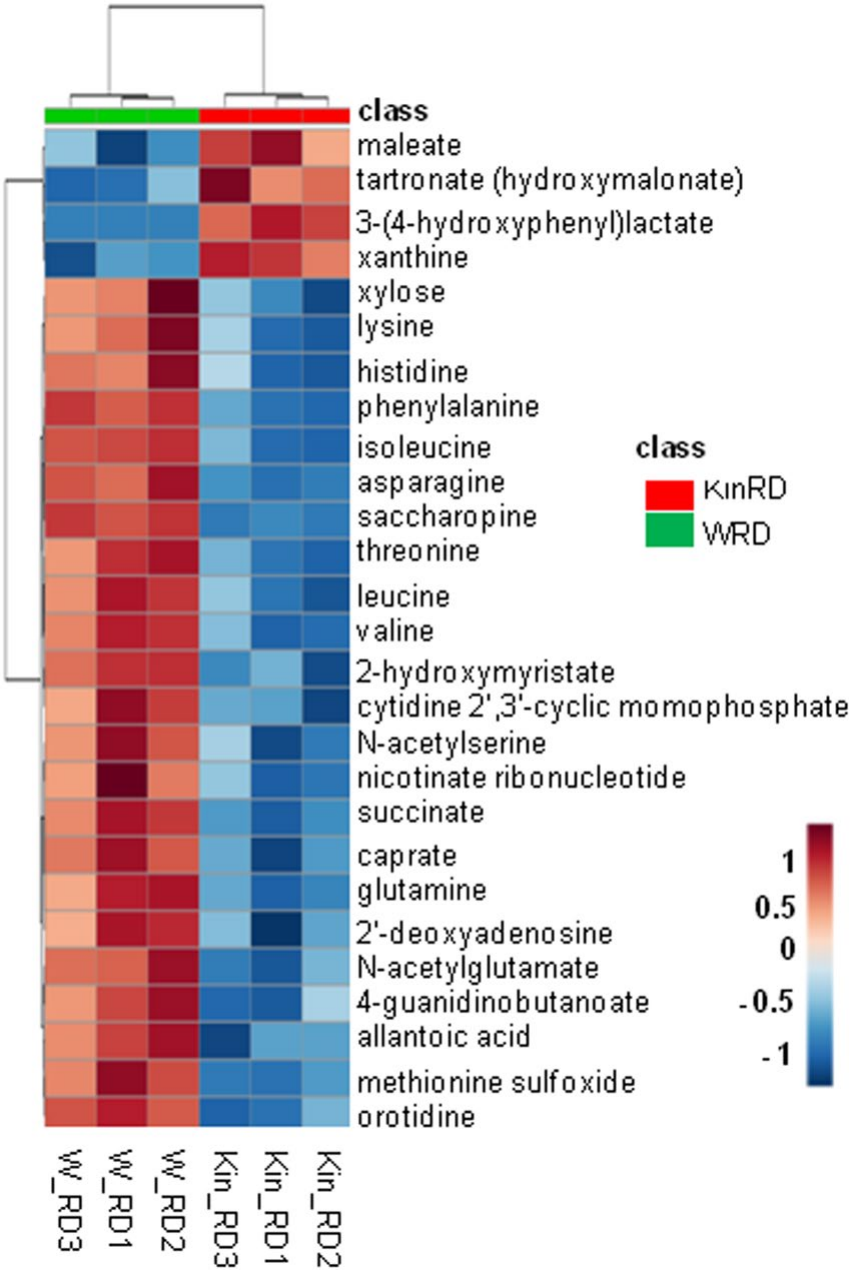
Hierarchical clustering (Euclidean distance measure and Ward clustering algorithm) of 27 metabolites with significant changes (*P* ≤ 0.05 and *q* ≤ 0.1) in response to kinetin treatment at radicle protrusion stage (RD). The color (from red to blue) represents the log-transformed raw scaled imputed value for each metabolite from high to low, respectively.

At the radicle protrusion stage, the majority of metabolites with significant fold changes induced by kinetin treatment belonged to the amino acid, nucleotide and carbohydrate super-pathways (Fig. 4). The most significant changes were noticed in the amino acid super-pathway, in which saccharopine (FC = 0.18) and 3-(4-hydroxyphenyl)lactate (FC = 3.51) were the metabolites with the highest variations in their levels in response to the treatment applied (Supplementary Dataset S1). As for the lipid metabolism, significant changes in the relative metabolite levels were observed for caprate (FC = 0.25). Interestingly, significant changes in relative levels of metabolites belonging to the nucleotide super-pathway were detected for cytidine 2′, 3′-cyclic monophosphate (FC = 0.5), orotidine (FC = 0.5) and xanthine (FC = 2.06). Significant changes were also noticed in the carbohydrate metabolism, namely regarding maleate (FC = 1.21) and tartronate (hydroxymalonate, FC = 1.42). Indeed, the heatmap generated (Fig. 5) with all the samples and the 27 differentially accumulated metabolites hierarchical clustering (Euclidean distance, Ward clustering algorithm) corroborated the earlier results obtained by PCA for samples collected at the radicle protrusion stage. The dendrogram shows that the replicates for each treatment cluster together, based on the changes observed in detected metabolites (Fig. 5). Also, the metabolites were grouped into two major clusters, one representing four metabolites significantly more accumulated in seeds imbibed with kinetin ((3–4-hydroxyphenyl)lactate, maleate, tartronate, xanthine) while another major cluster gathers the 23 metabolites significantly accumulated in water-imbibed seeds at the radicle protrusion stage. In summary, this highlights a significant reduction of the relative metabolite contents in kinetin-treated seeds when compared to untreated samples.

Quantitative enrichment analysis was also conducted over the 27 metabolites that revealed significant changes induced by the experimental conditions at the radicle protusion stage (KinRD *vs* WRD). These results evidenced the major metabolic pathways whose response was significantly triggered by the experimental conditions. The top and most significant metabolite sets (pathways) are lysine degradation (*P* = 0.000006, FDR = 0.0002), purine metabolism (*P* = 0.00005, FDR = 0.0008), phenylalanine and tyrosine metabolism (*P* = 0.0002, FDR = 0.0023), methionine metabolism (*P* = 0.0008, FDR = 0.0023) and valine/leucine/isoleucine degradation (*P* = 0.0009, FDR = 0.0023) (Supplementary Dataset S1). Based on the reported data, metabolomic analyses highlighted that most of the differentially accumulated metabolites were reduced at the radicle protrusion stage. Such results reflect a metabolite depletion resulting from faster germination, or a stress situation. However, the observed growth impairment in the kinetin-treated seeds leans towards the occurrence of stress conditions.

### Kinetin triggers genotoxic stress at the radicle protrusion stage

**Figure 6:**
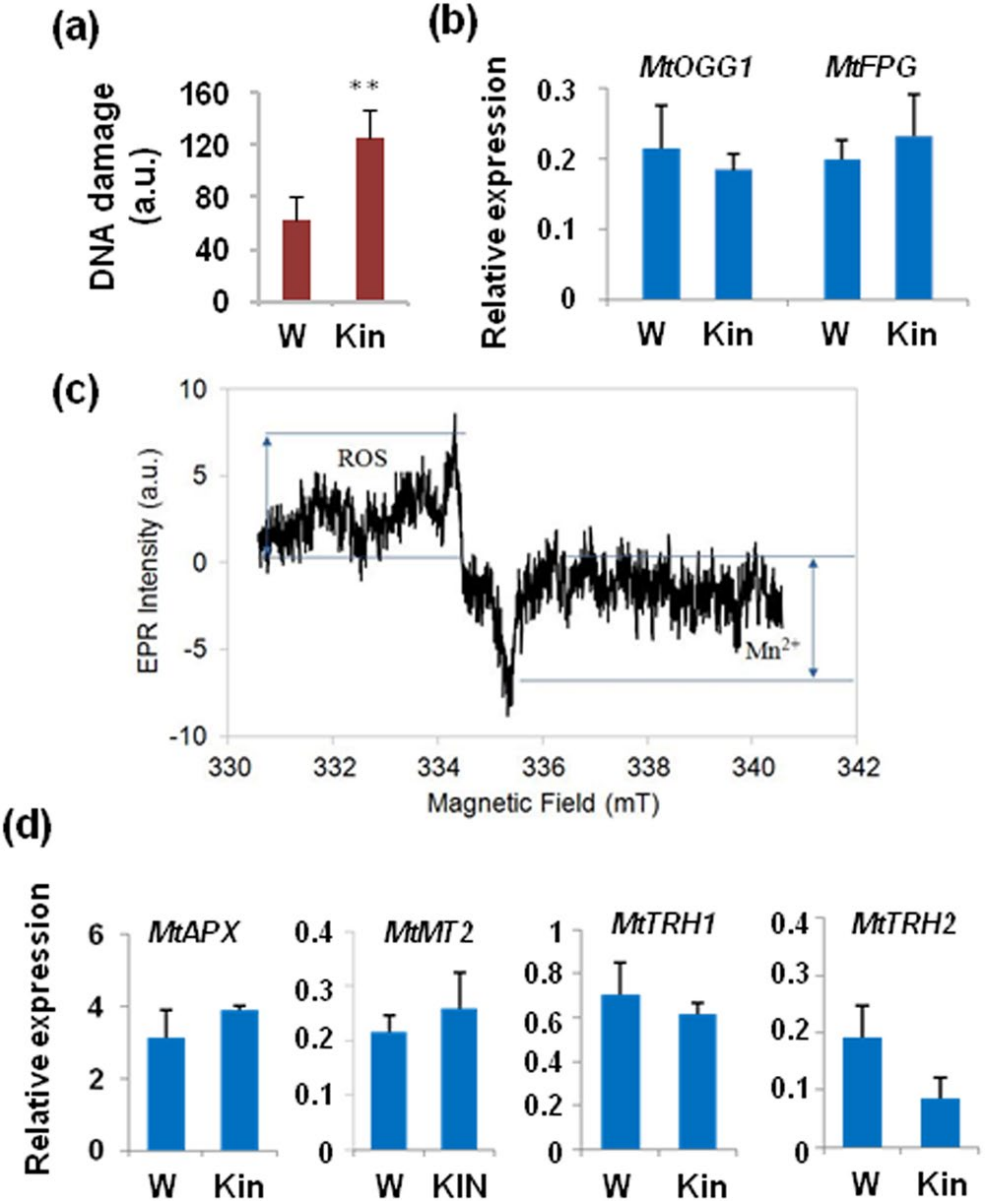
(**a**) Alkaline comet assay was used to measure the accumulation of DNA strand breaks in *M*. *truncatula* seeds collected at the radicle protrusion stage. (**b**) Transcript levels of *MtOGG1* and *MtFPG* genes in seeds collected at the radicle protrusion stage determined by *q*RT-PCR analyses. (**c**) EPR spectrum generated by kinetin-treated *M*. *truncatula* seeds collected at the radicle protrusion stage. The enlargement of the central part of the same EPR spectrum shows those regions corresponding to reactive oxygen species (ROS) and organic radicals, respectively. The EPR spectrum was recorded at low field. For the iron region (range from 100 to 550 mT) an applied microwave power of 10 mW with a modulation amplitude of 0.5 mT was used. For organic radicals and Mn^2+^, a range from 331 to 341 mT and an applied microwave power of 10 mW and a modulation amplitude of 0.2 mT were applied. (**d**) Transcript levels of *MtAPX*, *MtMT2*, *MtTRH1* and *MtTRH2* genes in seeds collected at the radicle protrusion stage determined by *q*RT-PCR analyses.Values are expressed as mean ± SD of three independent replications with 20 seeds for each replication. Asterisks indicate statistically significant differences determined using Student’s *t*-test (*P < *0.05). a.u., arbitrary units. W, control seeds imbibed with water. Kin, seeds imbibed with 0.5 mM kinetin. APX, ascorbate peroxidase. FPG, formamidopyrimidine-DNA glycosylase. MT2, type 2 metallothionein; OGG1, 8-oxoguanine DNA glycosylase.TRH, thioredoxin. mT, milliTesla.

DNA damage during early seed imbibition has been reported as a consequence of ROS accumulation during rapid water uptake^5,6,8^. Although it is still difficult to figure out the presence of metabolic signatures of genotoxic stress during seed germination, changes in nucleotide super-pathway were reported by Pagano *et al*.^13^ and linked to the occurrence of DNA damage induced by sodium butyrate. In the present work, significant changes in the nucleotide super-pathway were detected at the radicle protrusion stage, notably an increase in the levels of xanthine, an intermediate of purine degradation. Linked to xanthine overproduction, a study in animals pinpointed this metabolite as a hallmark of DNA damage in irradiated cells^35^. Hence, to investigate the levels of DNA damage in our system, an alkaline comet assay was carried out on radicles collected at the radicle protrusion stage. The results showed a significant (*P* = 0.009) increase in DNA strand breaks in Kin treatments (125.98 ± 20.67 a.u.), compared to W samples (62.89 ± 18.40 a.u.) (Fig. 6a). Additionally, a qRT-PCR analysis was carried out to monitor the expression profiles of specific genes with known roles in DNA damage repair. Nonetheless, no significant changes were detected in the expression of *MtOGG1* (*8-OXOGUANINE GLYCOSYLASE/LYASE*) and *MtFPG* (*FORMAMIDOPYRIMIDINE GLYCOSYLASE/LYASE*), genes involved in the removal of oxidative DNA lesions during imbibition^5,10,36^ (Fig. 6b). On the other hand, both *MtOGG1* and *MtFPG* transcripts were progressively accumulated during seed imbibition in W and Kin samples, respectively (Supplementary Table 2 and Supplementary Fig. 3), possibly revealing that DNA repair was required to the same extent, independently of treatments.

### Kinetin does not impact the free radical steady-state levels in *M*. *truncatula* seeds

Electron paramagnetic resonance (EPR) spectroscopy is a sensitive technique used for the qualitative and quantitative detection of free radical species in seeds^37^. To assess the free radical profile of *M*. *truncatula* seeds at the radicle protrusion stage, untreated and kinetin-treated seeds were frozen and the radical concentration was determined at 120 K. At this temperature, radicals are blocked inside the icy matrix (hence, stable) allowing their detection and quantification. For each sample, EPR analyses were performed at a low field to detect iron (Fe^3+^), while for the quantification of organic radicals and manganese (Mn^2+^) the measurements were centered on 225 mT. A representative example of the EPR spectrum generated in *M*. *truncatula* seeds imbibed with kinetin collected at the radicle protrusion stage is shown in Fig. 6c. The enlargement of the central part of the EPR spectrum allows to evidence regions corresponding to ROS and organic radicals, respectively. Since no spin traps were used, the measured quantity represents the free radical steady-state concentration. No significant differences were observed between EPR spectra generated by untreated and kinetin-treated seeds collected at the radicle protrusion stage, suggesting that the exogenous application of kinetin does not impact the free radical species profiles. It should be noticed that similar results were obtained with seeds collected at 2 h and 8 h of imbibition (data not shown). Overall, the reported data reflect the invariance of the steady-state concentration of radicals and show that the treatment with kinetin did not trigger ROS accumulation in *M*. *truncatula* seeds at the radicle protrusion phase. To further investigate the status of the antioxidant defence, a set of antioxidant genes (*MtAPX*, *ASCORBATE PEROXIDASE*; *MtMT*2, *TYPE* 2 *METALLOTHIONEIN*; *MtTRH1* and *MtTRH*2, *THIOREDOXIN*) that mark the pre-germinative metabolism^5,9,13,36^ was also investigated (Fig. 6d). No significant changes in *MtAPX* and *MtMT2* transcript levels were detected in Kin seeds at the radicle protrusion stage. On the other hand, *MtTRH1* and *MtTRH2* genes, encoding h-type thioredoxins that act as ROS scavengers to prevent oxidative damage upon imbibition^38^, were significantly down-regulated in Kin samples, compared to W control (Fig. 6d). The reduced antioxidant response well corroborated the results from the EPR analysis (Fig. 6c), showing that treatment with kinetin does not impact the free radical steady-state levels in *M*. *truncatula* seeds at the radicle protrusion stage. As previously observed for the DNA repair genes *MtOGG1* and *MtFPG*, for all the tested antioxidant genes the transcripts were progressively accumulated during seed germination in both W and Kin samples throughout imbibition (2 h and 8 h) until the radicle protrusion stage (Supplementary Table 2 and Supplementary Fig. 2), thus indicating that the antioxidant response triggered during the imbibition phase was somewhat sufficient to cope with any perturbation caused by the application of exogenous kinetin.

## Discussion

Despite the well-known role of cytokinins in promoting cell division^23,24^ and morphogenesis, very few studies have explored the potentialities of kinetin treatments to enhance seed vigor and germination in legumes^39^. Kinetin has been tested, alone or in combination with other phytohormones/chemicals to improve germination, however the limited number of reports so far available provide only a fragmentary picture of its potential use as priming agents compared to other phytohormones frequently used, as gibberellins^40^. To address this gap of knowledge, we investigated the effect of kinetin during *M*. *truncatula* seed germination to evaluate its suitability as priming agent in legumes. We found that the treatment with 0.5 mM kinetin, applied thorughout germination, was able to speed-up germination but caused radicle growth impairment. Using multidisciplinary analyses that combines untargeted metabolomics, EPR, and gene expression, we investigated the metabolites, along with ROS and gene profiles, that were disturbed during germination in response to 50 mM kinetin.

To our knowledge, this is the first report in legumes that exploits metabolomics to provide evidence of selected metabolites associated with exposure to kinetin and evaluates the effects of the phytohormone not only as growth inducer but also as stress agent. Our results showed that the Kin treatment accelerated the germination process, but the prolonged exposure to the phytohormone led to seedling growth impairment especially at the root level. Considering the impact that phytohormones (including cytokinins) can have on seed dormancy and germination^41^, we need to specify that the *M*. *truncatula* seeds used in this study represent freshly harvested material kept under dry storage conditions at room temperature for more than ten months, defined as after-ripening. Such treatment was reported to be effective in releasing dormancy and promoting seed germination in *M*. *truncatula*^42,43^. Independently of treatments, fully after-ripened seeds always germinated within two days from the beginning of imbibition, reaching a maximum germination percentage of 80%. This suggests that there was no physiological dormancy in the *M*. *truncatula* seed lot whereas the fraction (20%) of seeds that were not able to uptake water and failed to germinate might be associated with physical dormancy, a trait that follows distinct kinetics compared to those occurring during after-ripening^43^. Other parameters that can affect *M*. *truncatula* seed germination include temperature and light conditions^43^. Under our controlled experimental conditions, neither light conditions nor the applied temperature did not seem to affect the process since after-ripening increases the temperature range compatible with germination^43^. The 0.5 mM concentration was the lowest dose that accelerated germination, thus the most cost-effective considering the possibility of future applications for priming protocols. In our previous works on *M*. *truncatula*, we have characterised the temporal window from the beginning of imbibition until the radical protrusion stage in terms of antioxidant and DNA repair response by monitoring gene expression profiles under conditions that challenge germination^5,9,10,13,36^. This was done in the attempt to figure out the most informative timepoints of the pre-germinative metabolism to be selected for more in-depth investigations, namely ‘omics’. In the case of *M*. *truncatula* seeds challenged with the stress agents trichostatin A^36^ and sodium butyrate^13^, respectively, the timepoints corresponding to 2 h and 8 h of imbibition were found to be representative of the seed response in terms of significant up-regulation of antioxidant and DNA repair genes. These two informative timepoints were also maintained for investigating the impact of kinetin on the pre-germinative metabolism of *M*. *truncatula* seeds. According to the definition provided by Bewley^44^, the 2 h timepoint corresponds to the early imbibition status (stage I) while at the later timepoint (8 h) the seed is fully imbibed (early stage II).

The impact of the phytohormone on the seed metabolomic profiles resulted in significant changes only at the radicle protrusion stage and the overall reduction in metabolites levels, observed in kinetin-treated seeds when compared to water imbibed samples, can be regarded as a consequence of the accelerated degradation of the seed reserves required to support faster germination rate and fulfill the process^41^. Among the differentially accumulated compounds found at the radicle protrusion stage, 3-(4-hydroxyphenyl)lactate, member of the amino acid super-pathway, showed the highest variations in response to kinetin. This phenolic acid with ROS scavenging properties^45^ was also differentially accumulated in *M*. *truncatula* seeds under genotoxic stress conditions induced by the histone deacetylase inhibitor sodium butyrate^13^. It is possible that this metabolite represents a recurrent metabolic signature of the seed response to genotoxic stress as well as xanthine, also accumulated in response to kinetin at the radicle protrusion stage. It is worth noting that xanthine is a recurrent hallmark of DNA damage in irradiated cells, as revealed by studies on the radiation-responsive metabolome^35^. This suggests that changes in xhantine levels occurring during seed germination might be related to peaks in genotoxic damage, although in-depth studies will be necessary to further support such hypothesis. Differences were also revealed in UDP-glucose/UDP-galactose inositol polyphosphates with roles in sucrose and starch metabolism as well as in signaling^46,47^, agmatine and orotic acid, intermediates in polyamine and pyrimidine biosynthesis, respectively. Saccharopine, an intermediate in lysine degradation^48^ is also included in this set of metabolites. Protein turnover and amino acid degradation are also required at the onset of germination since the carbon skeletons of amino acids are directed into the TCA cycle whereas oxidation of lysine and other amino acids releases electrons that enter the mitochondrial electron transport chain. Overall, the profiles observed at 2 h and 8 h of imbibition, reflect the contribution of the different metabolic super-pathways to those essential physiological and biochemical changes that require *ex novo* biosynthesis or degradation to support germination. Moreover, when considering the possible changes observed during imbibition, it is worth to mention a relevant but not statistically significant accumulation of deoxycarnitine and 1-palmitoyl-2-linoleoyl-GP and depletion of 2-hydroxymyristate, 5-methylcytidine, and salicylate. All these metabolites, whose levels are perturbated in response to kinetin at 2 h of imbibition, deserve further investigation to assess their possible involvement in the seed response to kinetin-based priming.

Aside from the metabolite depletion, further proof that extended exposure to kinetin results in stress conditions is also given by the comet assay-based profiles of DNA damage that highlighted the overall genotoxicity induced by the exogenous application of the phytohormone (Fig. 6a). The genotoxic effects of cytokinins have been investigated in animals, where it was seen that exposure to high concentrations (500 nM) of kinetin induced significant DNA damage, as evidenced by a comet assay carried out on in HL60 cells, HaCaT human keratinocyte cells, NRK rat epithelial kidney cells and human peripheral lymphocytes^49^. In another study, Cabello *et al*.^50^ tested the effect of cytokinins and cytokinin nucleosides in various human cancer cell lines and observed that the accumulation of DNA damage was associated with rapid upregulation of DNA damage associated genes, e.g. *CDKN1A* (*CYCLIN DEPENDENT KINASE INHIBITOR 1A*) or *GADD153* (*GROWTH ARREST AND DNA DAMAGE-INDUCIBLE PROTEIN*). Nonetheless, under the experimental conditions of our study, the kinetin-induced DNA damage did not trigger significant up-regulation of the BER associated genes *MtOGG1* and *MtFPG*. This suggests that the BER pathway may not be required in this case, while the implication of other DNA repair pathways cannot be ruled out. Within this context, the cross-talk between DDR and cytokinins is still poorly explored even *in planta*, although there is evidence of interplay. For instance, Davis *et al*.^51^ demonstrated that double-strand breaks accumulation in *Arabidopsis* lateral root primordia not only triggers the SOG1(SUPPRESSOR OF GAMMA RESPONSE)-mediated up-regulation of DNA repair genes but also stimulates the expression of cytokinin biosynthesis/signaling genes leading to the inhibition of cell division. Kataya *et al*.^52^ also highlighted a link between DNA repair and cytokinins by investigating the *Arabidopsis PSY2L (PLATINUM SENSITIVE 2 LIKE)* mutant. Besides metabolite depletion and evident genotoxic injury, exposure to kinetin did not result in enhanced accumulation of free radical species nor into increased antioxidant gene expression, implying a global diminishment of the cellular metabolic activities. Indeed, other studies also evidenced that, although exogenously applied cytokinins could prevent senescence and positively impacted the plant antioxidant response^53^, the prolonged cytokinin exposure or high concentration resulted in impair growth^54^. It should be also noted that kinetin itself plays a role as ROS scavenger^55^ and that it has an anti-glycating activity that protects proteins against reactive aldehydes^56^. This may also be among the reasons why no changes in ROS profiles and antioxidant gene expression were observed under the Kin treatment in our experimental conditions, compared to control. On the other hand, DNA repair and antioxidant genes were upregulated in Kin-treated seeds throughout the tested timepoints with profiles similar to those recorded in W samples. Apparently, kinetin did not influence this specific aspect of seed metabolism

## Conclusions

In the present work, we demonstrate that the treatment of *M*. *truncatula* seeds with 50 mM kinetin applied throughout germination, accelerated radicle protrusion but causes radicle growth impairment. This shows the dual role of kinetin as a growth inducer and stress agent, likely due to the long exposure to the phytohormone. Seed metabolomics has provided a list of metabolites and metabolic pathways affected by kinetin during *M*. *truncatula* germination. Most of the changes observed were linked to the faster metabolic depletion associated with a faster germination. The study highlighted the differential accumulation of two metabolites, namely 3-(4-hydroxyphenyl)lactate and xanthine, potentially linked to stress. With additional and more targeted studies, these metabolites could be used to monitor stress-mediated events during germination and to figure out, for instance, the timepoint when priming treatments should be stopped to prevent toxicity towards seeds.

## Methods

### Plant materials, treatments, and phenotyping

For imbibition, *M*. *truncatula* seeds (Jemalong cultivar, kindly provided by Dr. Ana Barradas, Fertiprado L.d.a., Vaiamonte-Monforte, Portugal) were sown in Petri dishes (9 cm diameter) containing two filter papers moistened with 2.5 mL distilled water (control, W) or with 2.5 mL of 0.5 mM kinetin (6-furfurylaminopurine, Sigma-Aldrich, Milan, Italy) for phytohormone treatments. For germination tests, seeds were kept in a growth chamber at 22 °C under light conditions with a photon flux density of 150 μmol m^−2^ s^−1^, photoperiod of 16/8 h and 70–80% relative humidity. For each treatment, five independent replications with 20 seeds per replication were analyzed. Seeds with protrusion of the primary radicle were considered germinated. Germination percentage, scored along two consecutive days after imbibition, and the time to reach 50% germination (T_50_) were calculated as previously reported^57^. The time to obtain 50% germination (T_50_) was calculated according to the following formula of Coolbear *et al*.^58^, as modified by Farooq *et al*.^57^:

$${{\rm{T}}}_{50}={{\rm{t}}}_{{\rm{i}}}+\frac{(N/2-{{\rm{n}}}_{{\rm{i}}})({{\rm{t}}}_{{\rm{j}}}-{{\rm{t}}}_{{\rm{i}}})}{({{\rm{n}}}_{{\rm{j}}}-{{\rm{n}}}_{{\rm{i}}})}$$

where N is the final number of germinating seeds and n_j_ and n_i_ are the cumulative number of seeds germinated by adjacent counts at times t_j_ and t_i_, respectively, when n_i_ < N/2 < n_j_.

N values were annotated for each replicate until a plateau was reached, choosing the 10^th^ day as the standard timepoint to annotate them. The appearance of the first leaf on the resulting seedlings was scored during 14 days. Fresh and dry weights of imbibed seeds and seedlings were also measured. For molecular analyses, seeds were harvested at the indicated time points, namely 2 h and 8 h of imbibition with water or with 0.5 mM kinetin and at the post-germination stage (radicle protrusion; radicle length ≤ 3 mm). Due to the reduced T_50_ value observed in the phytohormone-treated seeds, the sampling procedure for the radicle protrusion stage was conducted earlier than for water-imbibed seeds. Kinetin-treated seeds were collected starting from the 25^th^ hour from the beginning of imbibition whereas water-treated seeds were collected subsequently, starting from the 40^th^ hour. All the samples were weighed and stored in liquid N_2_.

### Metabolomic profiling

Non-targeted metabolomic profiling was performed by Metabolon Inc. (Durham, NC, U.S.A.) (www.metabolon.com), based on three independent platforms: ultra-high-performance liquid chromatography/tandem mass spectrometry (UHLC/MS/MS^2^) optimized for basic species, UHLC/MS/MS^2^ optimized for acidic species, and gas chromatography/mass spectrometry (GC/MS), as described in Evans *et al*.^59^. Global metabolomic profiling was performed on methanol extracts from powdered frozen samples (100 mg) of seeds imbibed with water for 2 h (W2), 8 h (W8) and seeds at the radicle protrusion stage (W_RD). Samples from seeds imbibed with 0.50 mM kinetin for 2 h and 8 h (Kin2, Kin8), and seeds at the radicle protrusion stage (Kin_RD) were also profiled. Metabolomic analyses were conducted onto biological triplicates. Each biological replicate corresponds to a pool of at least 20 seeds. Data from seeds imbibed with water was previously described^13^ since the kinetin treatment was part of a larger experimental design. Metabolites were identified by automated comparison of the ion features of the experimental samples with a reference library of chemical standard entries that included retention time, molecular weight (m/z), preferred adducts, and in-source fragments as well as associated MS/MS2 spectra and curated by visual inspection for quality control using an in-house resources^59,60^. Raw area counts for each biochemical compound were normalized by dividing each sample value per sample fresh weight. Then, this value was rescaled by dividing each sample value by the median value for the specific biochemical. For missing data (nulls), the minimum observed values for that compound was imputed. Prior to data analysis, scaled imputed data was log transformed. Metabolites were also mapped onto general biochemical pathways, as provided in the Kyoto Encyclopedia of Genes and Genomes (KEGG) (www.genome.jp/kegg) and Plant Metabolic Network (PMN) (www.plantcyc.org/).

For metabolomic profiling, Welch’s two-sample *t*-tests were used to determine whether or not each metabolite had a significant change in abundance, focusing on comparisons between treated and non-treated samples for the same timepoint (Kin2 *versus* W2; Kin8 *versus* W8 and KinRD *versus* WRD). Prior to statistical computations, scaled imputed data were log transformed (natural logarithm). An estimate of false discovery rate for the list of all identified compounds was also provided, taking into account the multiple comparison tests conducted in metabolomic-based studies^61^. Metabolites that achieved statistical significance (*P* ≤ 0.05 and *q* ≤ 0.1) were considered differentially accumulated (DA) and selected for further analysis. Principal component analysis (PCA), quantitative enrichment analysis, heatmaps, and clustering analysis were conducted using the software resources available at Metaboanalyst 3.0 (www.metaboanalyst.ca) following user´s guide specifications^62^. While PCA was conducted over all identified metabolites, the remaining analyses were conducted only over DA metabolites present at least in one of the three comparisons studied.

### RNA extraction, cDNA synthesis, and quantitative real-time polymerase chain reaction

RNA isolation was carried out from water- and kinetin-imbibed *M*. *truncatula* seeds, respectively, collected at 2 h and 8 h (W2, Kin2; W8, Kin8) and seeds collected at the radicle protrusion stage (WRD, KinRD) as previously described^63^. cDNAs were obtained using the RevertAid First Strand cDNA Synthesis Kit (Thermo Fisher Scientific, Milan, Italy) according to the manufacturer’s suggestions. *q*RT-PCR was carried out with the Maxima SYBR Green qPCR Master Mix (2×) (ThermoFisher Scientific) according to supplier’s indications, using a Rotor-Gene 6000 PCR apparatus (Corbett Robotics Pty Ltd, Brisbane, Australia). Amplification conditions were as follows: denaturation at 95 °C for 10 min, and 45 cycles of 95 °C for 15 s and 60 °C for 60 s. Oligonucleotide primers were designed using the Real-Time PCR Primer Design program from GenScript (https://www.genscript.com/ssl-bin/app/primer) and validated through the online software Oligo Analyzer (https://eu.idtdna.com/calc/analyzer) (Supplementary Table 1). Sequences were retrieved using the NCBI gene database (https://www.ncbi.nlm.nih.gov/). For each oligonucleotide set, a no-template water control was used. Quantification was carried out using the *MtPDF2-PROTODERMAL FACTOR 2* (Medtr6g084690) as reference gene^64^ for the experimental conditions (treated versus untreated) used in this work. The raw, background-subtracted fluorescence data provided by the Rotor-Gene 6000 Series Software 1.7 (Corbett Robotics) was used to estimate PCR Efficiency (E) and threshold cycle number (C_t_) for each transcript quantification. The Pfaffl method^65^ was used for relative quantification of transcript accumulation and statistic analysis performed with REST2009 Software V2.0.13 (Qiagen GmbH, Hilden, Germany).

### Comet assay

For Comet assay analyses, water- and kinetin-imbibed seeds were collected at the radicle protrusion stage (WRD, KinRD). Kinetin-treated seeds were collected starting from the 25^th^ hour from the beginning of imbibition whereas water-treated seeds were collected subsequently, starting from the 40^th^ hour. Radicles were cut with a razor blade and immediately frozen in liquid N_2_. For each sample, 300 μL TE buffer (0.4 M Tris HCl pH 7.4, 1 mM EDTA) was added to the frozen tissues that were quickly and gently chopped with a sharp razor blade to release the nuclei as described by Macovei *et al*.^66^. For each treatment, two independent experiments were carried out with three independent replications (10 radicles per replication). Nuclei were extracted from *M*. *truncatula* radicles as reported^13^. The suspension containing the purified nuclei and a solution containing 1% low melting point agarose (Sigma-Aldrich) in phosphate-buffered saline (PBS) at 37 °C were mixed in equal volume. Aliquots (120 μL each) were pipetted onto agarose pre-coated slides and solidified on ice. Slides were incubated for 20 min at room temperature in high salt lysis buffer (2.5 M NaCl, 100 mM Tris-HCl pH 7.5, 100 mM EDTA) to disrupt the nuclear membrane. Nuclei were denatured in alkaline buffer (1 mM Na_2_EDTA, 300 mM NaOH, pH > 13) for 30 min at 4 °C and then electrophoresed in the same buffer for 25 min at 0.72 V cm^−1^ in a cold chamber. After electrophoresis, slides were washed twice in 0.4 M Tris-HCl pH 7.5 for 5 min, rinsed once in 70% ethanol (v/v) for 12 min at 4 °C and dried at room temperature overnight. Exposure to alkaline conditions causes DNA unwinding and visualization of strand breaks. Slides were stained with 20 μL DAPI (4′-6-Diamidine-2′-phenylindole dihydrochloride; 1 μg mL^−1^, Sigma-Aldrich). For each slide, one hundred nucleoids were scored, using a fluorescence microscope (Olympus BX51, Olympus Italia S.R.L.) with an excitation filter of 340–380 nm and a barrier filter of 400 nm. Nucleoids were classified and results were expressed in arbitrary units (a.u.) according to Collins^67^.

### Electron paramagnetic resonance

EPR analyses were carried out as reported^13^ on the water- and kinetin-treated seeds collected at 2 h, 8 h of imbibition and at the radicle protrusion stage. Seeds were transferred in quartz tubes, quickly frozen with liquid N_2_ and then stored in liquid N_2_. EPR spectra were recorded at 120 K with a Bruker EMX-10/12 spectrometer (Bruker BioSpin GmbH, Karlsruhe, Germany) operating in X-band, equipped with an ER4119HS cavity and a temperature controller. Two different instrument settings were used to better characterize iron and other radicals. As for the iron region (range from 100 to 550 mT), an applied microwave power of 10 mW with a modulation amplitude of 0.5 mT was used. For other radicals, a range from 331 to 341 mT and an applied microwave power of 10 mW and a modulation amplitude of 0.2 mT were applied. The intensity of the EPR signal was measured by using the peak height and normalized to seed weight, amplification gain and number of scans. The g-values were calculated by using a sample of Cr^3+^ dispersed in MgO (g = 1.9797) as a reference. For each treatment combination, two independent experiments with three replicated samples were carried out.

### Statistical analysis

Data from germination tests were analyzed using two-tailed heteroscedastic *t*-test focusing on the comparison between kinetin- and water-imbibed seeds collected at the same sampling time point. Means were considered statistically different when *P* ≤ 0.05. As for *q*RT-PCR data and comet assay analysis, statistically, significant differences were determined using Student’s *t*-test. Means were considered statistically different when *P* ≤ 0.05.

## Supplementary information

Supplementary Information

Supplementary Dataset S1
