# Supplementary material for: Metabolic signatures of germination triggered by kinetin in Medicago truncatula
Source: Sci Rep. 2019 Jul 18;9:10466. doi: 10.1038/s41598-019-46866-6 (PMC6639397; doi:10.1038/s41598-019-46866-6)
Supplement: Supplementary file 1 — Supplementary Information [file 41598_2019_46866_MOESM1_ESM.pdf]

Supplementary Information

Metabolic signatures of germination triggered by kinetin in *Medicago truncatula*

Susana de Sousa Araujo[1,#], Andrea Pagano[2,#] , Daniele Dondi[3], Simone Lazzaroni[3], Eduardo Pinela[1], Anca Macovei[2], Alma Balestrazzi[2,*]

[1] Instituto De Tecnologia Química e Biológica ITQB-UNL António Xavier (ITQB-NOVA) Avenida da Republica, Estação Agronómica Nacional 2780-157-Oeiras, Portugal

[2] Department of Biology and Biotechnology 'L. Spallanzani', via Ferrata 9, 27100 Pavia, Italy

[3] Department of Chemistry, Viale Taramelli 12, 27100 Pavia, Italy

[*] *Corresponding author:* AB, e-mail: alma.balestrazzi@unipv.it

[#] These authors equally contributed to the work

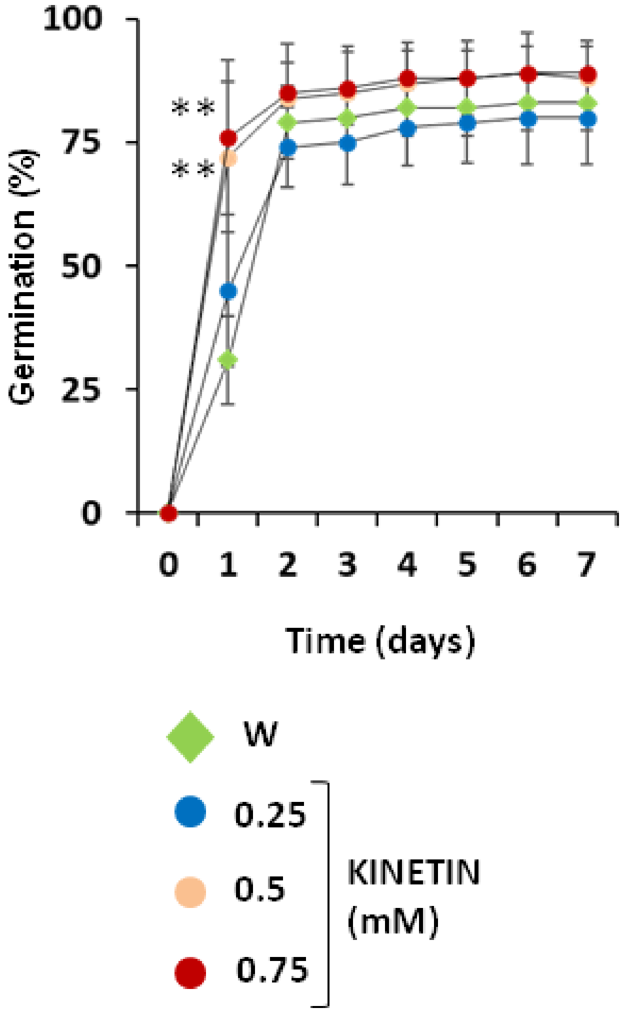

Supplementary Fig. 1. Kinetin anticipates germination in *M. truncatula*. Germination percentage of *M. truncatula* seeds imbibed with kinetin (0.25, 0.5 and 0.75 mM) and control (W) seeds imbibed with water. Values are expressed as mean ± SD of five independent replications with 20 seeds for each replication. Asterisks indicate statistically significant differences determined using Student's *t*-test.

Supplementary Table 1. Results of phenotyping analyses performed on *M. truncatula* seeds exposed to kinetin treatments. The T_{50} of seed germination is defined as the time required to reach 50% germination, calculated on the total number of germinating seeds counted on the 10th day after the beginning of imbibition. The T_{50} of the first leaves is defined as the time required to reach 50% of seedling displaying the first couple of true leaves, calculated on the total number of seedlings displaying second leaves at the 14th day after the beginning of imbibition. Values are expressed as mean ± SD of five independent replications with 20 seeds for each replication. Phenotyping analysis was also performed on four-day old seedlings developed in presence/absence of kinetin. Values are expressed as mean ± SD of three independent replications with 20 seeds for each replication. Asterisks indicate statistically significant differences determined using Student's *t*-test.W, control seeds imbibed with water.

Tissue/stage Parameter	***Treatment*** W	Kinetin (mM)		
		**0.25**	**0.5**	**0.75**
germination T_{50} [days]	1.2064 ± 0.1616	0.9246 ± 0.2354	0.6554 ± 0.1480***	0.6039 ± 0.1135 ***
first leaves T_{50} [days]	7.9833 ± 0.3699	5.7633 ± 0.1382 ***	5.9135 ± 0.2353***	5.7729 ± 0.1766 ***
Four-day old seedlings				
Fresh weight [mg/seedling]	35.2889 ± 1.5392	27.9000 ± 3.3198 *	32.6444 ± 0.9623	38.1778 ± 3.7185
Dry weight [mg/seedling]	2.3556 ± 0.0770	1.8333 ± 0.2186 *	2.1889 ± 0.0839	2.3222 ± 0.3533
Radicle length [mm]	20.0000 ± 1.0000	16.3333 ± 2.5166	11.6667 ± 0.5774 ***	10.0000 ± 1.0000 ***

(*) $P < 0.05$; (**) $P < 0.01$; (***) $P < 0.001$

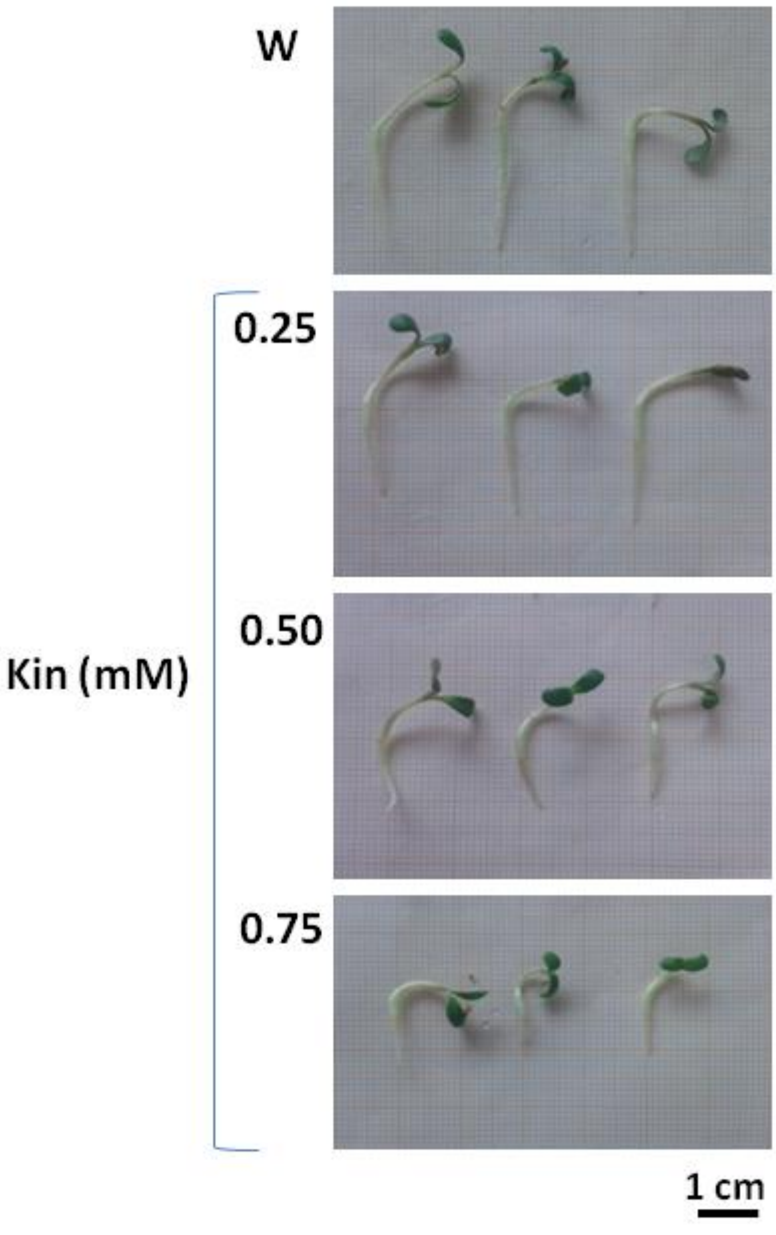

Supplementary Fig. 2. Phenotype of four-day-old *M. truncatula* seedlings developed from seeds imbibed with water (W) and increasing kinetin (Kin) doses (0.25, 0.5, and 0.75 mM).

Supplementary Table 2. Relative gene expression values measured in *M. truncatula* untreated seeds imbibed with water (W) and seeds treated with 0.5 mM kinetin (Kin) collected at 2 h and 8 h of imbibition and at the radicle protrusion stage. Values are means ± SEM (Standard Error of the Mean), n = 3 (replicates) per treatment group.

a-c = Means in a row without a common superscript letter differ ($P < 0.05$) as analyzed by two-way ANOVA and the TUKEY test (Assaad, H.I., Hou, Y., Zhou, L., Carroll, R.J. & Wu, G. 2015, Rapid publication-ready MS-Word tables for two-way ANOVA. *SpringerPlus* **4,** 33). *APX*, ascorbate peroxidase. *FPG*, formamido pyrimidine-DNA glycosylase. *MT2*, type 2 metallothionein. *OGG1*, 8-oxoguanine glycosylase/lyase. *TRXH*, h-type thioredoxin.

**Gene**	**2h**		**8h**		**RD**		***P*-value**		
	**W**	**Kin**	**W**	**Kin**	**W**	**Kin**	**Time**	**Treatment**	**T1×T2[1]**
MtAPX	0.0908 ± 0.0123^{c}	0.0671 ± 0.00801^{c}	1.44 ± 0.271^{b}	0.877 ± 0.0732^{bc}	3.11 ± 0.456^{a}	3.89 ± 0.0724^{a}	<0.001	0.724	0.031
MtMT2	0.0033 ± 0.00108^{b}	0.00519 ± 0.00145^{b}	0.0565 ± 0.0019^{b}	0.0303 ± 0.00204^{b}	0.216 ± 0.0178^{a}	0.259 ± 0.0376^{a}	<0.001	0.657	0.164
MtTRH1	0.23 ± 0.00854^{c}	0.0881 ± 0.0183^{c}	0.418 ± 0.0159^{b}	0.176 ± 0.0216^{c}	0.704 ± 0.085^{a}	0.616 ± 0.0308^{a}	<0.001	<0.001	0.182
MtTRH2	0 ± 0^{c}	0 ± 0^{c}	0.165 ± 0.0369^{ab}	0.0712 ± 0.0149^{bc}	0.193 ± 0.031^{a}	0.0845 ± 0.022^{bc}	<0.001	0.003	0.067
MtOGG1	0.0271 ± 0.0075^{c}	0.0219 ± 0.00681^{c}	0.0984 ± 0.00504^{bc}	0.0789 ± 0.0217^{c}	0.215 ± 0.0348^{a}	0.184 ± 0.0129^{ab}	<0.001	0.236	0.78
MtFPG	0.0304 ± 0.00489^{b}	0.0511 ± 0.0132^{b}	0.215 ± 0.025^{a}	0.0866 ± 0.0221^{b}	0.2 ± 0.0159^{a}	0.232 ± 0.0349^{a}	<0.001	0.178	0.005

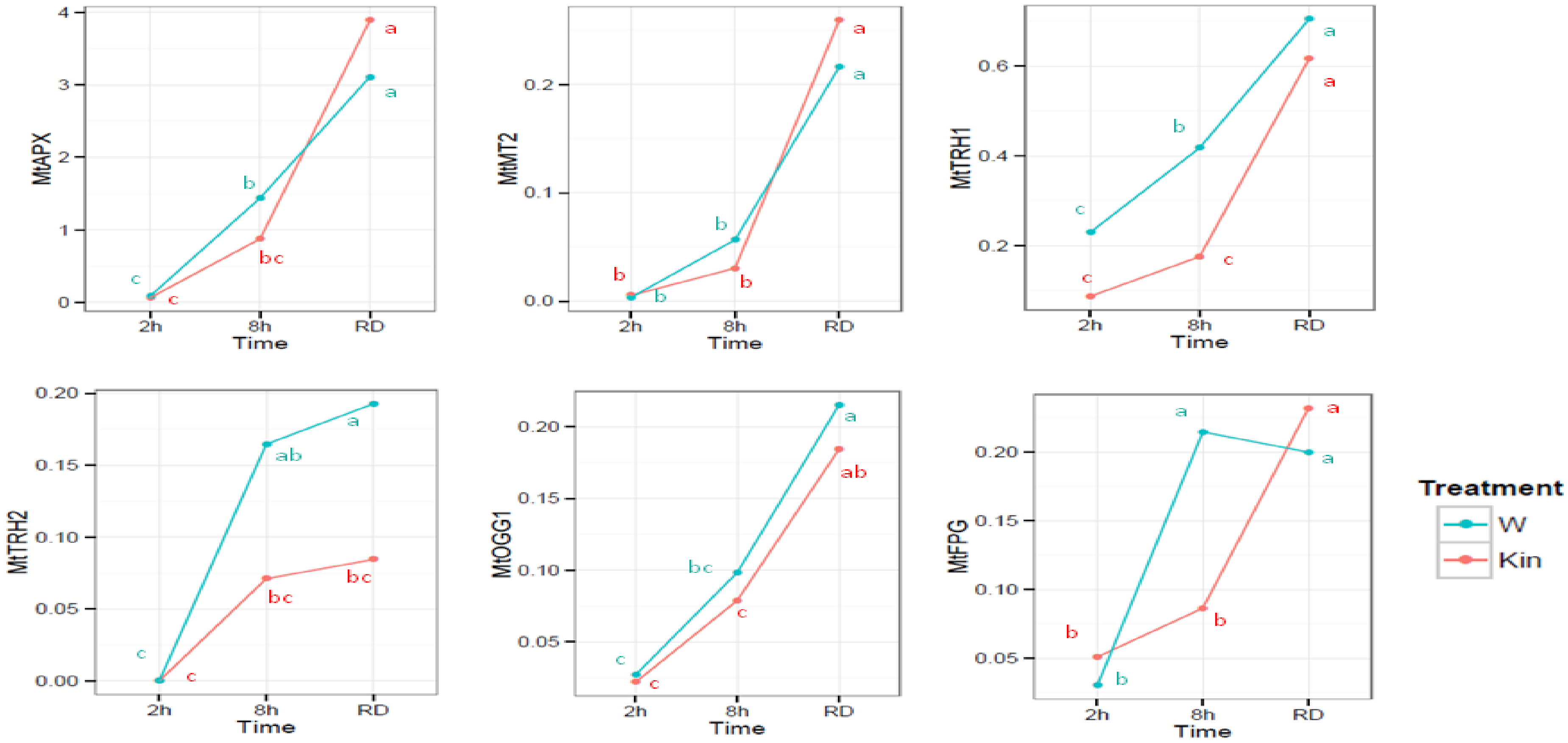

Supplementary Fig. 3. Relative gene expression values measured in *M. truncatula* untreated seeds imbibed with water (W) and seeds treated with 0.5 mM kinetin (Kin) collected at 2 h and 8 h of imbibition and at the radicle protrusion stage. Values are means ± SEM (Standard Error of the Mean), n = 3 (replicates) per treatment group. a-c = Means in a row without a common superscript letter differ ($P < 0.05$) as analyzed by two-way ANOVA and the TUKEY test (Assaad, H.I., Hou, Y., Zhou, L., Carroll, R.J. & Wu, G. 2015, Rapid publication-ready MS-Word tables for two-way ANOVA. *SpringerPlus* **4,** 33). *APX*, ascorbate peroxidase. *FPG*, formamido pyrimidine-DNA glycosylase. *MT2*, type 2 metallothionein. *OGG1*, 8-oxoguanine glycosylase/lyase. *TRXH*, h-type thioredoxin.

Supplementary Table 3. List of oligonucleotide primers used for *q*RT-PCR analyses. For each oligonucleotide set, PCR efficiency is reported. Quantification was carried out using *MtPDF2* as reference gene for the experimental conditions (treated versus untreated) used in this work**.** *APX*, ascorbate peroxidase. *FPG*, formamido pyrimidine-DNA glycosylase. *MT2*, type 2 metallothionein*OGG1*, 8-oxoguanine glycosylase/lyase. *TRXH*, h-type thioredoxin. *PDF*, protodermal factor.

**Gene (accession number)**	**Forward Primer**	**Reverse Primer**	**Efficiency**
MtAPX (Medtr4g061140)	5'-AGCTCAGAGGTTTCATCGCT-3'	5'-CGAAAGGACCACCAGTCTTT-3'	1.76
MtFPG (Medtr2g126800)	5’-TCCTTTCAATTCGGTATGGC-3’	5’-GCTCCAAACCATCGTCTAGC-3’	1.81
MtMT2 (Medtr8g060850)	5'-CATGTCAAGCTCATGCGGCAAC-3'	5'-TGCCGTAGTTGTTTCCCTTCCC-3'	1.72
MtOGG1 (Medtr3g088510)	5'-AAACACCGCACCTTCTCAAT-3'	5'-TGTGGAGATGTTTGAGGGAA-3'	1.73
MtTRXH1 (Medtr3g112410)	5’-ATGGCAGCAGAAGAGGGACA-3’	5’-TTGGGGCAATGAAACGGCAC-3’	1.81
MtTRXH2 (Medtr1g023140)	5’-ATGGTCTGTTGAGGCTATGC-3’	5’-CATCCTTATGCTTGGTGATTGC-3’	1.88
MtPDF2 (Medtr6g084690)	5'-GTGTTTTGCTTCCGCCGTT-3'	5'-CCAAATCTTGCTCCCTCATCTG-3'	1.78
